# HIV viral suppression at different thresholds and duration of treatment in the dolutegravir treatment era in Sierra Leone: a nationwide survey

**DOI:** 10.1186/s12985-023-02245-2

**Published:** 2023-11-29

**Authors:** Jin-Wen Song, Guang Yang, Matilda N. Kamara, Wei Sun, Qun Guan, Umu Barrie, Darlinda F. Jiba, Abdulai Tejan Jalloh, Ming Liu, Francis K. Tamba, George A. Yendewa, Ligui Wang, Rongtao Zhao, Sulaiman Lakoh

**Affiliations:** 1 Senior Department of Infectious Diseases, the Fifth Medical Center of PLA General Hospital, Beijing, China; 2 Department of Clinical Laboratory, the Fifth Medical Center of PLA General Hospital, Beijing, China; 3 Tropical Infectious Disease Prevention and Control Center, Freetown, Sierra Leone; 4 College of Medicine and Allied Health Sciences, University of Sierra Leone, Freetown, Sierra Leone; 5 School of Public Health, Southern Medical University, Guangzhou, China; 6 The Fifth Medical Center of PLA General Hospital, Beijing, China; 7 Infectious Disease Research Network, Freetown, Sierra Leone; 8 Ministry of Health and Sanitation, Government of Sierra Leone, Freetown, Sierra Leone; 9 Department of Medicine, Case Western Reserve University School of Medicine, Cleveland, OH USA; 10 Division of Infectious Diseases and HIV Medicine, University Hospitals Cleveland Medical Center, Cleveland, OH USA; 11 Johns Hopkins Bloomberg School of Public Health, Baltimore, MD USA; 12 Chinese PLA Center for Disease Control and Prevention, Beijing, China; 13 Sustainable Health Systems Sierra Leone, Freetown, Sierra Leone

**Keywords:** Viral load, Viral suppression, Viral suppression rate, Low-level viremia, Dolutegravir

## Abstract

**Introduction:**

Viral load assessment for people living with HIV is key for monitoring treatment and achieving the 95-95-95. In this study, we aimed to assess the degree of viral suppression at different thresholds and treatment duration after the introduction of dolutegravir-based therapy in ten public hospitals in Sierra Leone.

**Methods:**

We used a cross-sectional study design to recruits patients aged 18 years or older between August 2022 and January 2023. Statistical analyses were performed using R-software. Logistic regression was used to assess factors independently associated with viral suppression. The level of significance was set at P < 0.05.

**Results:**

Of the 2,253 patients recruited, 1,720 (76%) were women and 1,705 (76%) were receiving a fixed dose combination of tenofovir, lamivudine and dolutegravir. The median age and duration of anti-retroviral therapy (ART) was 36.0 (IQR, 28.0–45.0) years and 40.9 (IQR, 14.4–79.6) months, respectively. Using a threshold of HIV RNA < 1000 copies/mL, 1,715 (88.4%) patients on ART for more than 6 months were virally suppressed. Viral suppression rates were higher with dolutegravir-based (1,277, 89.5%) than efavirenz-based (418, 86.2%) ART. HIV RNA was < 200 copies/mL in 1,643 (84.6%) patients or < 50 copies/mL in 1,487 (76.6%) patients or between 50 and 999 copies/mL in 228 (11.7%) patients. Viral suppression rates at different ART durations (months) were as follows: 84.2% (≤ 3), 88.8% (4–6), 90.9% (6–12), and 88.1% (> 12). Viral suppression rates were higher for patients aged 40 or older (40–50 years: aOR 2.05, 95%CI 1.41–3.04, P < 0.01; 50–60 years: aOR 2.51, 95%CI 1.53–4.35, P < 0.01; >60 years: aOR 2.69, 95%CI 1.28–6.63, P = 0.02). Men had 49% lower odds of viral suppression than women (aOR 0.50, 95% CI 0.38–0.67, P < 0.01).

**Conclusion:**

We report a viral suppression rate of 88.4% among patients on treatment for at least 6 months, with higher rate of suppression with dolutegravir than efavirenz. Factors associated with virological suppression were age and gender, emphasizing the need for innovative differentiated ART delivery models to optimize viral suppression and achieve the 95% target.

## Introduction

A fast-track strategy to end the HIV epidemic calls for low and middle-income countries to adopt the Joint United Nations Agency on HIV/AIDS (UNAIDS) 95-95-95 global target; 95% of people living with HIV (PLHIV) should know their status, 95% of those who know their status should receive antiretroviral treatment (ART) and 95% of those in care should have a suppressed viral load by 2030. The World Health Organization (WHO) recommends viral load assessment for all PLHIV as a key intervention for treatment monitoring in PLHIV and achieving the third 95 [2]. However, the WHO-defined viral suppression threshold of < 1,000 RNA copies/mL underestimates adverse outcomes such as subsequent virologic failure and resistance mutations in patients with low-level viremia of 50–999 copies/mL [3, 4]. These observations have prompted calls for a review of the < 1000 copies/mL WHO threshold for viral suppression to prevent the increasing burden of HIV-drug resistant mutations in sub-Saharan Africa [5–8]. Consequently, in its July 2021 guidelines, WHO recommend enhanced adherence counselling and a repeat of HIV viral load testing after 3 months in patients with a viremia of > 50 to ≤ 1000 copies/mL, similar to patients with a viremia > 1,000 copies/mL [2].

Sierra Leone is a low HIV prevalence setting with a national seroprevalence of 1.7% in 2019. However, the country faces several challenges in its response to the HIV epidemic, including a high prevalence of advanced HIV diseases, late-stage diagnosis, and opportunistic fungal and non-fungal infections [9–13]. Prior to the introduction of dolutegravir in Sierra Leone, HIV viral suppression rate among PLHIV receiving ART for at least 6 months was 64.6% when applying the WHO threshold of < 1,000 copies/mL [14]. As a result, the National AIDS Control Program of Sierra Leone switched from the use of non-nucleoside reverse transcriptase inhibitor-based regimen as the preferred drug of choice to the use of a dolutegravir-based regimen as the preferred first-line ART in 2020.

Since the introduction of regimen transition, viral load assessment has been challenging, and it is unclear what effect dolutegravir-based therapy has on viral suppression. Understanding the effect of dolutegravir-based therapy on different HIV viral thresholds at different treatment durations has important policy implications, as it provides data to understand the implications of lowering the viral threshold on the scarce resources of low-income countries [15]. In this study, we aimed to assess the degree of viral suppression using different thresholds and different duration of ART after the introduction of dolutegravir-based therapy in ten public hospitals in Sierra Leone.

## Methods

### Study design and study population

We used a cross-sectional study design to collect primary data from adult patients aged 18 years or older between August 2022 and January 2023.

### Study setting

Sierra Leone is a low-income country in West Africa with five geographical regions, such as the Western Area and North, East, South and Northwestern regions with a population of 7 million in 2015. About 22% (1.5 million) of its population live in the Western Area [16]. The health system is divided into tertiary, secondary and primary care. While tertiary care is the highest level of care in Sierra Leone, secondary care services are basic medical, surgical, maternal and child health services that are provided at district hospitals. Primary care is the lowest level of care and is provided by peripheral health units, including community health centers (CHC).

At the time of the study, there were 439 sites reporting data to the Sierra Leone National AIDS Control Program, of which 215 sites accounted for just 2% of the more than 58,000 PLHIV receiving ART by April 2022. There are 72 large sites where at least 100 PLHIV are receiving ART, accounting for 86% of all PLHIV receiving ART in the country.

We selected health facilities where at least 800 PLHIV were receiving treatment by April 30, 2022 at a time when the study is being planned and conducted the study in 10 of these public health facilities. The health facilities and their PLHIV populations included Connaught Hospital (3,187), Waterloo Community Health Center (2,999), Rokupa Government Hospital (2,392), Jenner Wright Clinic (1,341), Princess Christian Maternity Hospital (1,101), Lumley Government Hospital (988) and 34 Military Hospital (804) in the Western Area, Bo Government Hospital (2,679) in the South, Kenema Government Hospital (2,374) in the East, and Makeni Government Hospital (2,036) in the North. Two centers in the East (Koidu Government Hospital and Well Body Clinic) and two in the Western Area (Wellness Clinic and UMC Urban Center) with at least 800 PLHIV were excluded because of the distance or delay in obtaining consent to conduct the study. Connaught Hospital, Kenema Government Hospital, Makeni Government Hospital, Bo Government Hospital and 34 Military Hospital are tertiary/regional hospitals, while Rokupa and Lumley Government Hospitals are secondary hospitals. The remaining two facilities (Jenner Wright Clinic and Waterloo Community Health Center) are primary health facilities.

### Participants selection and laboratory procedure

After providing written informed consent, we recruited participants non-randomly as they presented to the different health facilities between August 2022 and January 2023. Demographic and HIV information were collected from patients using a standardized data collection form and cross-checked with clinical records.

Approximately 10 mL of venous blood was collected aseptically in EDTA vacutainer tubes and analyzed centrally at the Infectious Disease Prevention Center of the 34 Military Hospital in Freetown, Sierra Leone. The plasma was separated by centrifugation and stored at -80 °C until use. The HIV RNA was extracted using the TGuide S32 Magnetic Viral DNA/RNA Kit (Tiangen Biotech, Beijing, China) and the HIV viral load was determined by HIV-RNA Quantification Kit (Sansure Biotech Inc., Hunan, China) according to the manufacturer’s instructions. Amplification of HIV-1 RNA and standards were detected by the probes labeled with FAM channel and Select VIC/HEX/YELLOW channel to detect HIV Internal Control. HIV-1 RNA amplification and quantification were performed with the CFX96 real-time PCR detection system (Bio-Rad Laboratories, Hercules, CA, USA). Thermal cycling conditions used were 95 °C for 1 min for pre-denaturation and enzyme activation; 60 °C for 30 min reverse transcription; 95 °C for 1 min for cDNA pre-denaturation; 95 °C for 15 s for denaturation, and 58 °C for 30 s for annealing, extension and fluorescence collection followed by 45 cycles. The threshold for HIV RNA detection is 15 copies/mL.

### Data management and analysis

Statistical analyses were performed using the R-software version 4.2.3 (R Core Team, Vienna, Austria). Baseline characteristics were summarized using frequencies and medians. The differences in patient characteristics were assessed using Pearson’s Chi-squared test, Fisher’s exact test and Kruskal-Wallis H test. Multivariable binary logistic regression analysis was used to assess factors independently associated with viral suppression. The level of significance was P < 0.05.

## Results

### Demographic characteristics and HIV details

Over the course of the study, 2,253 patients were recruited. Their median age was 36.0 (IQR, 28.0–45.0) years. More than a third were under 30 years (758, 33.6%) and nearly half were married (1,080, 47.9%). Most were women (1,720, 76.3%), had been on ART for more than 24 months (1,434, 63.6%) and were receiving a fixed dose combination of tenofovir, lamivudine and dolutegravir (1705, 75.7%) (Table 1). The median duration of ART was 40.9 (IQR, 14.4–79.6) months.

**Table 1:** Demographic and HIV details of participants

		Type of facility				
Demographic/HIV details	Overall N (%)	Primary N (%)	Secondary N (%)	Tertiary N (%)		p-value
**Age (years)**						0.20
(0,30]	758 (33.6%)	209 (34.8%)	66 (35.1%)	483 (33.0%)		
(30,40]	693 (30.8%)	187 (31.1%)	70 (37.2%)	436 (29.8%)		
(40,50]	467 (20.7%)	116 (19.3%)	34 (18.1%)	317 (21.7%)		
(50,60]	245 (10.9%)	61 (10.1%)	13 (6.9%)	171 (11.7%)		
(60,100)	90 (4.0%)	28 (4.7%)	5 (2.7%)	57 (3.9%)		
**Sex**						0.01
Female	1,720 (76.3%)	486 (80.9%)	141 (75.0%)	1,093 (74.7%)		
Male	533 (23.7%)	115 (19.1%)	47 (25.0%)	371 (25.3%)		
**Marital status**						
Divorced	41 (1.8%)	13 (2.2%)	1 (0.5%)	27 (1.8%)		
Married	1,080 (47.9%)	310 (51.6%)	86 (45.7%)	684 (46.7%)		
Separated	69 (3.1%)	31 (5.2%)	10 (5.3%)	28 (1.9%)		
Single	795 (35.3%)	170 (28.3%)	68 (36.2%)	557 (38.0%)		
Widowed	268 (11.9%)	77 (12.8%)	23 (12.2%)	168 (11.5%)		
**ART regimen**						
TLD	1,705 (75.7)	311 (51.7)	136 (72.3)	1,258 (85.9)		
TLE	512 (22.7)	282 (46.9)	50 (26.6)	180 (12.3)		
ALD	2 (0.1)	0 (0.0)	0 (0.0)	2 (0.1)		
ALE	1 (< 0.1)	0 (0.0)	0 (0.0)	1 (< 0.1)		
Others	33 (1.6)	8 (1.3)	2 (1.1)	23 (1.5)		
**ART duration (months)**						0.10
(≤ 3)	196 (8.7%)	64 (10.6%)	20 (10.6%)	112 (7.7%)		
(3–6]	116 (5.1%)	32 (5.3%)	11 (5.9%)	73 (5.0%)		
(6–12]	186 (8.3%)	50 (8.3%)	19 (10.1%)	117 (8.0%)		
(12–24]	321 (14.2%)	67 (11.1%)	24 (12.8%)	230 (15.7%)		
(> 24)	1,434 (63.6%)	388 (64.6%)	114 (60.6%)	932 (63.7%)		

*TLD: tenofovir + lamivudine + dolutegravir; TLE: tenofovir + lamivudine + efavirenz*

*ALD: abacavir + lamivudine + dolutegravir; ART: antiretroviral therapy*

*ALE: abacavir + lamivudine + efavirenz*

### HIV viral suppression

Overall, 1983 (88.0%) had an HIV RNA level < 1000 copies/mL, regardless of the duration of ART (Table 2). Using a viral threshold of < 1000 copies/mL, 1,715 (88.4%) patients who had received ART for more than 6 months were virally suppressed (Table 3). The rate of viral suppression was higher in dolutegravir-based therapy (1,277, 89.5%) than in efavirenz-based therapy (418, 86.2%) (p = 0.002). There were 1,643 (84.6%) patients with a HIV RNA < 200 copies/mL or 1,487 (76.6%) with a viral load < 50 copies/mL. Low-level viremia (viral load between 50 and 999 copies/mL) was reported in 228 (11.7%) patients (Table 3).

**Table 2:** Viral suppression at different threshold, regardless of duration thresholds disaggregated by level of care

Viral load (copies/mL)	Overall, N = 2253		Primary, N = 601		Secondary, N = 188		Tertiary, N = 1,464		p-value
	N	%, 95%CI	N	%, 95%CI	N	%, 95%CI	N	%, 95%CI	
< 1000	1,983	88.0 (86.6, 89.3)	511	85.0 (81.9, 87.7)	172	91.5 (86.3, 94.9)	1,300	88.8 (87.0, 90.3)	0.017
< 400	1,927	85.5 (84.0, 86.9)	487	81.0 (77.6, 84.0)	167	88.8 (83.2, 92.8)	1,273	87.0 (85.1, 88.6)	< 0.001
< 200	1,878	83.4 (81.7, 84.9)	477	79.4 (75.9, 82.5)	162	86.2 (80.2, 90.6)	1,239	84.6 (82.7, 86.4)	0.008
< 50	1,672	74.2 (72.3, 76.0)	434	72.2 (68.4, 75.7)	141	75.0 (68.1, 80.9)	1,097	74.9 (72.6, 77.1)	0.4
< 20	1,517	67.3 (65.3, 69.3)	398	66.2 (62.3, 70.0)	124	66.0 (58.7, 72.6)	995	68.0 (65.5, 70.3)	0.7

**Table 3:** Viral suppression using different thresholds disaggregated by regimen for patients taking ART for > 6 months

Viral load (copies/mL)	Overall	Dolutegravir-based regimen		Efavirenz-based regimen		Others		p-value
	N = 1,941	N = 1,427	95% CI	N = 485	95% CI	N = 29	95% CI	
< 1000	1,715 (88.4)	1,277 (89.5)	87.8, 91.0	418 (86.2)	82.7, 89.1	20 (69.0)	49.0, 84.0	0.002
< 400	1,680 (86.6)	1,256 (88.0)	86.2, 89.6	405 (83.5)	79.8, 86.6	19 (65.5)	45.7, 81.4	< 0.001
< 200	1,643 (84.6)	1,223 (85.7)	83.8, 87.5	402 (82.9)	79.2, 86.1	18 (62.1)	42.4, 78.7	0.002
< 50	1,487 (76.6)	1,098 (76.9)	74.7, 79.1	375 (77.3)	73.3, 80.9	14 (48.3)	29.9, 67.1	0.001
< 20	1,359 (70.0)	995 (69.7)	67.3, 72.1	350 (72.2)	67.9, 76.1	14 (48.3)	29.9, 67.1	0.022
≥ 50, < 1000	228 (11.7)	179 (12.5)	10.9, 14.4	43 (8.9)	6.6, 11.8	6 (20.7)	8.71, 40.3	0.023

CI: confidence interval; ART: antiretroviral therapy

Viral suppression rates were 84.2% for those who had been on ART for 3 months or less, 88.8% for those who had been on ART for 4 to 6 months, and 90.9% for those who had been on ART for 6 to 12 months. The viral suppression rate was 88.1% in patients receiving ART for more than 12 months (Table 4).

**Table 4:** Viral suppression at different threshold disaggregated by duration of ART and level of service delivery

Duration of ART (months)	Viral load (copies/mL)	Overall, N = 2253		Primary, N = 601		Secondary, N = 188		Tertiary, N = 1,464		p-value
		N	%, 95%CI	N	%, 95%CI	N	%, 95%CI	N	%, 95%CI	
(≤ 3)	< 1000	165	84.2 (78.1, 88.8)	56	87.5 (76.3, 94.1)	16	80.0 (55.7, 93.4)	93	83.0 (74.5, 89.2)	0.6
	< 400	148	75.5 (68.8, 81.2)	47	73.4 (60.7, 83.3)	14	70.0 (45.7, 87.2)	87	77.7 (68.6, 84.8)	0.7
	< 200	136	69.4 (62.3, 75.7)	43	67.2 (54.2, 78.1)	12	60.0 (36.4, 80.0)	81	72.3 (62.9, 80.2)	0.5
	< 50	100	51.0 (43.8, 58.2)	32	50.0 (38.1, 61.9)	9	45.0 (23.8, 68.0)	59	52.7 (43.1, 62.1)	0.8
	< 20	84	42.9 (35.9, 50.1)	24	37.5 (26.0, 50.5)	7	35.0 (16.3, 59.1)	53	47.3 (37.9, 56.9)	0.3
(3,6]	< 1000	103	88.8 (81.3, 93.7)	29	90.6 (73.8, 97.5)	10	90.9 (57.1, 99.5)	64	87.7 (77.4, 93.9)	> 0.9
	< 400	99	85.3 (77.3, 91.0)	28	87.5 (70.1, 95.9)	10	90.9 (57.1, 99.5)	61	83.6 (72.7, 90.9)	> 0.9
	< 200	99	85.3 (77.3, 91.0)	28	87.5 (70.1, 95.9)	10	90.9 (57.1, 99.5)	61	83.6 (72.7, 90.9)	> 0.9
	< 50	85	73.3 (64.1, 80.9)	27	84.4 (66.5, 94.1)	8	72.7 (39.3, 92.7)	50	68.5 (56.4, 78.6)	0.2
	< 20	74	63.8 (54.3, 72.4)	22	68.8 (49.9, 83.3)	7	63.6 (31.6, 87.6)	45	61.6 (49.5, 72.6)	0.8
(6,12]	< 1000	169	90.9 (85.5, 94.4)	43	86.0 (72.6, 93.7)	18	94.7 (71.9, 99.7)	108	92.3 (85.5, 96.2)	0.4
	< 400	169	90.9 (85.5, 94.4)	43	86.0 (72.6, 93.7)	18	94.7 (71.9, 99.7)	108	92.3 (85.5, 96.2)	0.4
	< 200	165	88.7 (83.0, 92.7)	42	84.0 (70.3, 92.4)	18	94.7 (71.9, 99.7)	105	89.7 (82.4, 94.4)	0.4
	< 50	148	79.6 (72.9, 85.0)	38	76.0 (61.5, 86.5)	17	89.5 (65.5, 98.2)	93	79.5 (70.8, 86.2)	0.5
	< 20	130	69.9 (62.7, 76.3)	33	66.0 (51.1, 78.4)	12	63.2 (38.6, 82.8)	85	72.6 (63.5, 80.3)	0.6
> 12	< 1000	1,546	88.1(86.5, 89.6)	383	84.2 (80.4, 87.3)	128	92.8 (86.7, 96.3)	1,035	89.1 (87.1, 90.8)	0.005
	< 400	1,511	86.1 (84.4, 87.7)	369	81.1 (77.1, 84.5)	125	90.6 (84.1, 94.7)	1,017	87.5 (85.5, 89.3)	0.001
	< 200	1,478	84.2 (82.4, 85.9)	364	80.0 (76.0, 83.5)	122	88.4 (81.6, 93.0)	992	85.4 (83.2, 87.3)	0.011
	< 50	1,339	76.3 (74.2, 78.3)	337	74.1 (69.7, 78.0)	107	77.5 (69.5, 84.0)	895	77.0 (74.5, 79.4)	0.4
	< 20	1,229	70.0 (67.8, 72.2)	319	70.1 (65.6, 74.2)	98	71.0 (62.6, 78.3)	812	69.9 (67.1, 72.5)	> 0.9

CI: confidence interval; ART: antiretroviral therapy

### Factors influencing viral suppression

Using a simplified multivariate analysis of data from all the 2,253 PLHIV using a stepwise backward procedure to eliminate non-significant factors (Table 5), older age (40–50 years: aOR 2.05, 95%CI 1.41–3.04, P < 0.01; 50–60 years: aOR 2.51, 95%CI 1.53–4.35, P < 0.01; >60 years: aOR 2.69, 95%CI 1.28–6.63, P = 0.02) had a higher viral suppression rate (HIV RNA < 1000 copies/mL). Compared with female patients, male patients had 50% lower odds of viral suppression (aOR 0.5, 95% CI 0.38–0.67, P < 0.01). Secondary (aOR 1.96, 95%CI 1.14–3.57, P = 0.02) and tertiary (aOR 1.39, 95%CI 1.02–1.89, P = 0.03) ART services were more likely to result in viral suppression than ART services in primary health care.

**Table 5:** Factors associated with viral suppression at a threshold of < 1000 copies/mL

Variable	Overall, N = 2,253	Suppressed (n, %) N = 1,983	aOR	95%CI	P-value
**Age**					
(0,30]	758	646 (85.2)	1.00	NA	
(30,40]	693	604 (87.2)	1.34	0.98–1.82	0.07
(40,50]	467	424 (90.8)	2.05	1.41–3.04	< 0.01
(50,60]	245	226 (92.2)	2.51	1.53–4.35	< 0.01
(60,100]	90	83 (92.2)	2.69	1.28–6.63	0.02
**Sex**					
Female	1,720	1,539 (89.5)	1.00	NA	
Male	533	444 (83.3)	0.50	0.38–0.67	< 0.01
**ART regimen**					
TLD	1,705	1,519 (89.1)	1.00	NA	
TLE	512	440 (85.9)	0.87	0.63–1.20	0.40
Others	36	24 (66.7)	0.24	0.12–0.52	< 0.01
**Hospital**					
Primary	601	511(85.0)	1.00	NA	
Secondary	188	172 (91.5)	1.96	1.14–3.57	0.02
Tertiary	1,464	1,300 (88.8)	1.39	1.02–1.89	0.03

ART: antiretroviral therapy; CI: confidence interval; aOR: adjusted odd ratio;

*TLD: tenofovir + lamivudine + dolutegravir; TLE: tenofovir + lamivudine + efavirenz*

## Discussion

Using an HIV RNA threshold of < 1000 copies/mL, we observed an overall viral suppression rate of 88.0% in all PLHIV, regardless of the duration of ART and 88.4% in PLHIV treated with ART over 6 months, regardless of regimen type and level of care. A similar viral suppression rate of 87.4% was reported in a national survey in South Africa [17]. In contrast, the rate of viral suppression in our population was higher than the 76.4% reported by PLHIV in Guinea-Bissau and the 40.9% reported in Ethiopia [18, 19]. The reasons for the higher viral suppression rate in Sierra Leone than other countries are unclear, but could be related to the introduction of dolutegravir-based regimen to the Sierra Leone treatment program in 2020, which may not be the case in Ethiopia and Guinea Bissau [18, 19]. Dolutegravir-based regimens are effective in reducing viral load and optimizing response to treatment [20, 21]. A recent study in Nigeria reported a viral suppression rate of 97.6% in patients using dolutegravir based therapy [22]. In our study, viral suppression to < 1000 copies/mL after 6 months of treatment was higher in the dolutegravir-based regimen than the efavirenz-based regimen (89.5 vs. 86.2%). Thus, we recommend that our HIV program maximize the impact of dolutegravir-based therapy by addressing known barriers, such as limited psychosocial counseling and challenges with virologic management, to optimize viral suppression to achieve the 95% national and global target [1, 14].

In the WHO and Sierra Leone guidelines, HIV viral load is recommended for all patients receiving ART for more than 6 months, and a viral load < 1000 copies/mL defines the threshold for viral suppression [2, 23]. The viral suppression rate using this threshold for patients who had received ART for 3 months or less in this study was 84.2%, higher than the 64.6% reported after 6 months of ART in the pre-dolutegravir era, suggesting that dolutegravir may influence earlier viral suppression than the 6 months conventional period [14, 21]. Viral suppression in patients on ART between 6 and 12 months is higher than viral suppression in patients taking ART for more than 12 months in our study. Although there is no direct explanation for the decline in the rate of viral suppression with the duration on ART, it could probably be due to the recent transitioning of ART regimen from a predominantly NNRTI-based regimen to dolutegravir-based regimen or perhaps it could be explained by the impact of the COVID-19 pandemic on treatment adherence in PLHIV staying long on ART and reflect on the need to strengthen adherence counselling, psychosocial support and other HIV services during public health emergency [24].

Between 2010 and 2016, a follow up study involving 927 sero-discordant gay couples with regular condomless anal sex by HIV-positive partner with viral loads < 200 copies/mL, and their HIV negative partner reported zero transmission of HIV, reinforcing the message of ‘undetectable equals untransmittable (U = U)’ and emphasizes the benefits of early HIV diagnosis and treatment [25]. A viral suppression of < 200 copies/mL in 84.6% of patients who have received ART for more than 6 months in our study warrants the introduction of U = U campaigns in Sierra Leone to reduce HIV-related stigma and communicate the scientific evidence that the transmission of HIV through sex or even breastfeeding is non-existent in PLHIV with an undetectable viral load [26, 27]. Moreover, unlike the WHO recommended threshold of < 1000 copies/mL to define viral suppression, the International Association of Providers of AIDS Care recommend a viral threshold of < 200 copies/mL to define virologic suppression, reinforcing the fact that our study has added to the evidence needed to support global advocacy to lower the threshold for viral suppression [3, 28]. It is important to note that low-level viremia of 50–999 copies/mL can cause virologic failure and inherent mutations that lead to HIV drug resistance and poor treatment response [27, 29, 30]. In our study, 11.7% of patients receiving ART for more than 6 months exhibited low level viremia. As per the WHO guidelines, patients with low-level viremia should receive enhanced adherence counselling and undergo a repeat viral load testing three months later [2]. Establishing functional viremia clinics, supported by the sustained availability of viral load testing services, is needed for the effective implementation of this recommendation in Sierra Leone.

Factors associated with suppressed viral load < 1000 copies/mL are older age, female sex and level of service delivery. Similar findings were reported in adolescents in Uganda, where age and duration of antiviral therapy are associated with virologic suppression [31]. In Ethiopia and South Africa, duration of antiviral treatment is significantly associated with virologic suppression in children and adults living with HIV, respectively [32, 33]. To address these inherent challenges, HIV programs should employ innovative differentiated ART models for close monitoring and improved care.

Our study has limitations. First, the study did not assess the status of adherence to ART in the different facilities and therefore has no information on the impact of the treatment interruptions on HIV viral suppression. HIV drug resistance and baseline CD4 count may be important predictors of viral suppression but were not assessed due to insufficient resources. The study was only conducted at large sites, and the results may differ from smaller facilities, where staff may be less well trained and care may be different. Finally, we plan to recruit 20% of PLHIV from each of the 10 sites between August 2022 and January 2023, but this plan was not achieved in Waterloo Community Health Center and Kenema, Bo and Makeni Government Hospitals due to the slow flow of patients. Thus, of the 19,901 PLHIV receiving ART in all these facilities as of April 30, 2022, only 2,253 (11.3%) were enrolled.

Nonetheless, this study provides national insights into the state of HIV viral suppression and proffer actionable recommendations on viral load management in Sierra Leone and other countries facing similar challenges.

## Conclusion

We report a viral suppression rate of 88.4% among PLHIV treated with ART for at least 6 months, with higher rate of suppression in dolutegravir than efavirenz-based therapy. Common factors associated with virological suppression were age and gender, emphasizing the need for innovative differentiated ART service delivery models to optimize viral suppression and achieve the national target of 95%.

## Data Availability

The data for this study is available at Fifth Medical Centre of PLA General Hospital, Beijing, China and Ministry of Health of Sierra Leone and will be made available upon request.
